# The Burden of Cold Agglutinin Disease on Patients’ Daily Life: Web-Based Cross-sectional Survey of 50 American Patients

**DOI:** 10.2196/34248

**Published:** 2022-07-22

**Authors:** Florence Joly, Lisa Anne Schmitt, Patricia Ann McGee Watson, Emilie Pain, Damien Testa

**Affiliations:** 1 Health Economics And Outcomes Research Sanofi Chilly-Mazarin France; 2 Public Affairs and Patient Advocacy Sanofi Genzyme Cambridge, MA United States; 3 Cold Agglutinin Disease Foundation Cherry Hill, NJ United States; 4 Carenity Paris France; 5 Moona Paris France

**Keywords:** autoimmune diseases, fatigue, perception, rare diseases, surveys and questionnaires, cold agglutinin disease, cold autoimmune hemolytic anemia

## Abstract

**Background:**

Cold agglutinin disease (CAD) is a rare disorder, affecting 15% of patients with autoimmune hemolytic anemia. Few studies have assessed CAD symptoms and their impact on daily life, but these studies did not address the patients’ perspectives.

**Objective:**

The aims of this study were to increase the knowledge about CAD through a patient-centric survey and to gain a better understanding of the burden of this disease.

**Methods:**

We conducted an internet-based survey in September 2020 among American patients registered on the CAD Unraveled website and members of the Cold Agglutinin Disease Foundation.

**Results:**

A total of 50 respondents were included in this study. Totally, 90% (45/50) of the patients reported having experienced fatigue. Fatigue was mainly reported on a daily basis, and approximately one-third of these patients (13/45, 29%) said that their fatigue was constant throughout the day. It has also been shown that CAD has a great impact on patients’ physical well-being, emotional well-being, social life, and household finances. The disease varies over time, with or without symptoms. A total of 88% (44/50) of the patients reported previous episodes of the increased intensity or sensitivity of their CAD symptoms, with a mean of 4.5 (SD 5.4) episodes reported during the past year. More than half of the patients (27/50, 54%) considered their disease to be moderate or severe, and 42% (21/50) of the study group reported that their symptoms had worsened since the time of diagnosis.

**Conclusions:**

Our study has provided new data on CAD symptoms, particularly data on the importance and type of fatigue and the fluctuation of CAD symptoms.

## Introduction

### Background

Autoimmune hemolytic anemias (AIHAs) are rare and heterogeneous disorders characterized by the destruction of red blood cells by warm or cold antibodies, thereby causing anemia and other related health issues [1-4]. AIHA is classified into three categories: warm, cold, and mixed [1,5,6]. Warm AIHA is characterized by the binding of polyclonal immunoglobulin (often immunoglobulin G) to red blood cell antigens (Rh proteins or glycophorins A-D). This binding is referred to as *warm* in that it occurs at most temperatures but is maximal at 37 °C [4,5]. Cold agglutinin disease (CAD) is the most common form of cold AIHA, accounting for 15 to 25% of AIHA cases [5-7]. CAD is recognized by the presence of immunoglobulin M autoantibodies, also known as cold agglutinins, which are active and cause hemolysis at cold temperatures, usually 3 °C to 4 °C [5,7]. This cold AIHA is composed of CAD, formerly known as primary CAD, and cold agglutinin syndrome, formerly known as secondary CAD [7]. There is no known cause for CAD. Cold agglutinin syndrome is associated with underlying conditions such as infection, malignancy, or immune disease [4,7].

CAD is a rare disease. Berentsen et al [8] suggested variations between cold and warm climates, reporting an incidence of 9 cases per million people per year in North Italy and 2 cases per million people per year in Norway, with a prevalence of 50 per million people in Italy and 200 per million people in Norway. An incidence of 1.8 cases per million person-years was reported based on the Danish national patient register [4]. CAD primarily affects middle-aged to older individuals; however, the disease has been observed in people aged as young as 30 years [7,9-11]. Some studies have indicated that women are slightly more affected by the disease than men [9].

Diagnosis of CAD is established with hemolytic anemia, reticulocytosis, hyperbilirubinemia, elevated lactate dehydrogenase, and positive Coombs test for anti-C3d and classically negative anti– immunoglobulin G [9]. After the test findings suggest CAD, the antibody titer and thermal activity should be determined to prevent overdiagnosis, because most agglutinins are clinically insignificant [9].

Most people with CAD have symptoms of hemolytic anemia such as paleness, shortness of breath, rapid heart rate, fatigue, weakness, dark urine, or pain [6,9,12]. Many people with CAD also experience pain and bluish coloring of the hands and feet (acrocyanosis) or Raynaud disease owing to slow or poor blood circulation [6,9,12]. CAD symptoms can vary throughout the course of a patient’s illness, involving fluctuation in CAD severity [7]. Anemia in CAD is often mild (hemoglobin level >10 g/dL) to moderate (hemoglobin level between 8 and 10 g/dL) and, in some cases, fully compensated, but it may be severe (hemoglobin level <8 g/dL) [13].

Importantly, febrile illness, trauma, and surgery can exacerbate hemolytic anemia [7,14], which can increase the risk of thromboembolic events and death [15]. Patients with mild anemia and minimal circulatory symptoms typically do not require specific pharmacological treatment [4,6,13]. These patients are advised to avoid exposure to cold [6,13], to limit the intensity of acrocyanosis and risk of developing trophic disorders. Moreover, any bacterial or viral infection should be treated [6].

Seasonal variation of these symptoms has been reported [16]. Symptoms were reported to be more severe when the patient is exposed to cold temperatures. This can lead to delay in diagnosis during warm periods.

At the time of this study, there was no approved treatment for CAD. However, patients with moderate anemia and hemoglobin levels below approximately 10 g/dL or disabling cold-induced circulatory symptoms may require blood transfusions. Rituximab monotherapy or rituximab along with bendamustine are recommended as first-line therapy for patients with severe symptoms, depending on individual patient characteristics [6,17]. Corticosteroid treatment is not recommended for patients with CAD, especially for long-term treatment [6]. Novel pharmacological treatment options are also currently under development to improve clinical management [13,17] and consequently, the quality of life of patients [4].

In addition to these treatments, patients are recommended to avoid cold. Mild CAD symptoms may be managed by avoiding exposure to cold temperatures, avoiding cold food and water, using room heaters, and wearing warm clothing (warm shoes, scarves, gloves, earmuffs, warm inners, and stockings) [17].

### Objectives

To enhance diagnosis and clinical management, it is necessary to better understand the symptom severity and impact of CAD from the patient’s perspective. Qualitative studies on CAD are scarce and lacking. Su et al [12] found from qualitative interviews of 16 patients with CAD that the most frequently reported symptoms were fatigue, tiredness, or lack of energy and reaction to cold environments [2,12]. The rarity of CAD limits the ability to perform large-scale studies. The aims of this study were to increase the knowledge about CAD through a patient-centric survey and gain a better understanding of the burden of this disease from patients living with CAD in the United States.

## Methods

### Study Design and Participant Recruitment

This study included qualitative and quantitative research using a web-based questionnaire. The recruitment period started on September 1, 2020, and lasted for less than a month. Invitations to complete a self-administered questionnaire and follow-up emails were sent to members of the CAD Unraveled website, a website created by Sanofi and dedicated to providing support, information, and tools to patients with CAD to help them manage their condition. The Cold Agglutinin Disease Foundation (CADF), a nonprofit foundation dedicated to educating and supporting patients living with CAD, also shared the survey with its members. The study was based on voluntary and free participation of patients who agreed to participate in the survey. The study’s inclusion criteria were as follows: patients self-reporting a diagnosis of CAD, aged ≥18 years, residing in the United States, and being a registered member of the CAD Unraveled website or CADF.

### Data Collection

The questionnaire (39 closed-ended questions and 5 open-ended questions) was designed by Carenity, in collaboration with Sanofi and CADF. The questionnaire was written in English, and the average estimated time needed to answer was 30 minutes.

The questionnaire started with a set of questions about the respondent’s profile (eg, age, gender, place of residence, and profession), CAD characteristics, and disease status (eg, disease duration, age at diagnosis, type of CAD, symptoms experienced, and episodes experienced). CAD episodes were defined as a few hours during which a patient is affected by severe CAD symptoms.

The second part of the questionnaire was related to patient’s perception of CAD severity and progression.

The third part assessed the patient’s expectations of CAD-related fatigue management. Distinction between fatigue or tiredness, weakness, and lack of stamina was provided. Fatigue was defined as the feeling of tiredness or exhaustion or the need to rest because of lack of energy or strength and the need for extra sleep. Weakness was considered as a lack of physical or muscle strength and the feeling that extra effort is required to move arms, legs, or other muscles. Finally, lack of stamina was defined as an inability to maintain or sustain prolonged physical effort or activity.

The fourth part of the questionnaire evaluated the management methods used at the time of the survey, previous management methods, and patient satisfaction regarding management methods used at the time of the survey.

Finally, the last part explored the patient’s perception of the CAD burden and the impact that CAD symptoms had on the patient’s daily life, professional activities, and household finances (eg, impact owing to health care costs and work absenteeism). CAD burden was assessed at three time points (eg, when usual or regular symptoms were experienced, when symptom intensity or sensitivity increased or new symptoms occurred, and when no symptoms were experienced).

### Statistical Analysis

Categorical variables are expressed as absolute frequencies and percentages. Continuous variable data are presented as mean (SD) for normal distribution and as median for nonnormal distribution. Narratives from open-ended questions were manually analyzed by identifying themes and subcategories.

Data processing and analysis were performed using RStudio (version 3.5.0; RStudio, Inc). Excel (Microsoft Corporation) 2013 was used to analyze the open-ended questions. The participants’ personal information is not publicly available owing to privacy laws.

### Ethics Approval

Written informed consent was obtained from all participants through information notice. The study protocol and patient materials were reviewed by the Western and Copernicus Group Institutional Review Board (formerly, New England Institutional Review Board; approval number 20202393).

## Results

### Description of the Study Population

**Figure 1:**
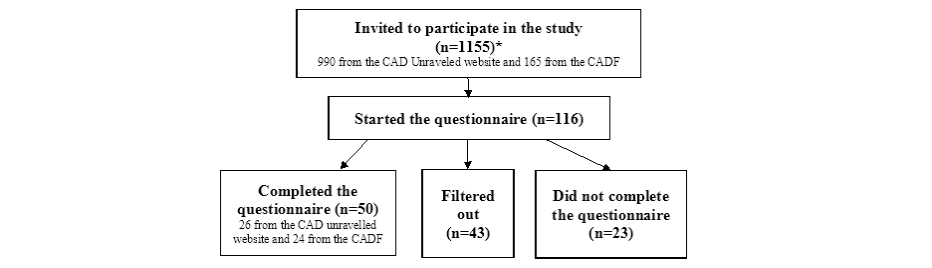
Disposition of survey respondents. CAD: cold agglutinin disease; CADF: Cold Agglutinin Disease Foundation. *Indicates people who had agreed to receive invitations to participate in the study.

A total of 116 participants started to fill in the questionnaire (registered as living in the United States or who had not indicated their country of residence), with 50 (43.1%) participants fulfilling the inclusion criteria and completing the questionnaire by September 24, 2020 (Figure 1). Patients were excluded from the study mainly because they indicated not living in the United States or not having CAD diagnosed by a health care professional.

Participants were located throughout the United States, with few participants from the Midwest. The mean age at enrollment was 66.7 (SD 8.5) years, and the men-to-women sex ratio was 0.22. Regarding employment status, 76% (38/50) of the patients declared that they were not working at the time of the survey and 20% (10/50) of the patients were working (Table 1).

At diagnosis, patients were on average aged 59.2 (SD 8.5) years. The main signs that led to diagnosis were blood tests (44/50, 88%) and presence of symptoms (26/50, 52%). Most patients (44/50, 88%) experienced symptoms before the diagnosis of CAD. The most frequently reported symptoms included fatigue (fatigue, tiredness, lack of stamina, or weakness; 37/50, 74%), shortness of breath (19/50, 38%), and acrocyanosis (18/50, 36%)

Among the 88% (44/50) of the patients who had experienced symptoms before being diagnosed, 34% (15/44) patients were diagnosed ≥2 years after their initial symptoms (Table 2). The delay between the development of initial symptoms and diagnosis was primarily owing to unawareness that the symptoms were associated with a disease (14/44, 32%) or because the patient had consulted multiple physicians before being properly diagnosed (13/44, 30%). The sociodemographic and clinical characteristics of the study participants are summarized in Tables 1 and 2.

**Table 1:** Sociodemographic characteristics of patients with cold agglutinin disease in the United States recruited for the web-based survey (N=50).

Sociodemographic characteristics		Values
**Sex, n (%)**		
	Women	41 (82)
	Men	9 (18)
**Age at the time of the survey (years), mean (SD; range)**		66.7 (8.5; 45-86)
	≤60, n (%)	12 (24)
	61-65, n (%)	5 (10)
	66-70, n (%)	14 (28)
	71-75, n (%)	13 (26)
	>75, n (%)	6 (12)
**Employment status^a^ at the time of the survey, n (%)**		
	Work—full-time	6 (12)
	Work—part-time	4 (8)
	Do not work	38 (76)
	Disabled	2 (4)

^a^Multiple-choice question.

**Table 2:** Clinical characteristics of patients with CAD^a^ in the United States recruited for the web-based survey (N=50).

Medical profile			Values
**Type of CAD (according to patients’ knowledge), n (%)**			
	Primary CAD	25 (50)	
	Secondary CAD	6 (12)	
	Do not remember	3 (6)	
	Do not know	16 (32)	
**Time since CAD diagnosis (years), mean (SD; range)**			7.5 (7.4; 1-30)
	<5, n (%)	21 (42)	
	5-10, n (%)	8 (16)	
	11-15, n (%)	5 (10)	
	>15, n (%)	7 (14)	
**Age at CAD diagnosis (years), mean (SD; range)**			59.2 (8.5; 41-76)
	<55, n (%)	17 (34)	
	55-60, n (%)	9 (18)	
	61-65, n (%)	13 (26)	
	>65, n (%)	11 (22)	
**Symptoms experienced before diagnosis (top 5)^b^, n (%)**			
	Fatigue or tiredness	37 (74)	
	Decreased stamina	23 (46)	
	Increased weakness	22 (44)	
	Shortness of breath	19 (38)	
	Acrocyanosis^c^	18 (36)	
Number of symptoms experienced before diagnosis, mean (SD)			4.6 (3.5)
**Interval between initial symptoms and diagnosis^d^, n (%)**			
	<6 months	15 (34)	
	6 months-1 year	7 (16)	
	1-2 years	6 (14)	
	2-3 years	7 (16)	
	>3 years	8 (18)	
	Do not remember	1 (2)	

^a^CAD: cold agglutinin disease.

^b^Multiple-choice question; complete list is given in Figure S1 in Multimedia Appendix 1.

^c^Cold and blue limbs.

^d^Sample size: n=44.

### CAD Symptoms Experienced and Perception of Disease Severity and Progression

More than half of the patients (27/50, 54%) considered their disease to be moderate or severe (Table 3). Patients with a severe form were principally affected by severe symptoms and were not satisfied with the efficiency of their management methods. Of the 50 patients, 31 (62%) patients reported that their CAD has progressed since their diagnosis: 21 (42%) patients thought that it has worsened and 10 (20%) thought that it has improved. Respondents who had experienced worsening of their CAD symptoms indicated that it occurred when the intensity or sensitivity of their symptoms increased (8/21, 38%), when new CAD symptoms appeared (4/21, 19%), and when both CAD symptom intensity or sensitivity increased and new CAD symptoms appeared (2/21, 10%). CAD varied over time: 88% (44/50) of the patients experienced episodes with increased intensity and sensitivity of CAD symptoms, 72% (36/50) experienced episodes with new CAD symptoms, and 54% (7/50) experienced episodes without symptoms. A total of 40% (20/50) of the patients had experienced all 3 types of episodes. Among the 94% (47/50) patients who reported CAD episodes with increased intensity or sensitivity or new CAD symptoms, the most cited symptoms experienced during a CAD episode were fatigue (fatigue, tiredness, lack of stamina, or weakness; 42/47, 89%) and shortness of breath (31/47, 66%; Table 3). In total, 94% (44/47) of the patients identified at least one factor that caused an increase in symptom intensity and sensitivity. Among the 13 factors cited, the three major triggers were cold temperature (39/47, 83%), winter (32/47, 68%), and air conditioning (26/47, 55%; Table 3).

Totally, 90% (45/50) of the patients reported having experienced fatigue (fatigue, tiredness, lack of stamina, or weakness). Among these 45 patients, a total of 40 (89%) patients reported the symptoms at the time of the survey. In total, 47% (21/45) of the participants reported that it was moderate, 29% (13/45) reported that it was mild, and 13% (6/45) reported that it was severe. Fatigue was reported on a daily basis by 44% (20/45) of the patients, several times a week by 27% (12/45), and only after physical exertion by 16% (7/45) of the patients. When fatigue was experienced, 31% (14/45) of the patients mentioned that it usually fluctuated during the course of the day, 29% (13/45) reported that fatigue was constant throughout the day, and 22% (10/45) reported that fatigue symptoms were more intense in the afternoon.

**Table 3:** Episodes of CAD^a^ reported by patients in the United States recruited for the web-based survey (N=50).

Episodes of CAD		Values
**Perception of CAD severity, n (%)**		
	Mild	16 (32)
	Moderate	21 (42)
	Severe	6 (12)
	Do not know	7 (14)
**Progression of the disease, n (%)**		
	Worsened	21 (42)
	Same	15 (30)
	Improved	10 (20)
	Do not know	4 (8)
**Number of CAD episodes experienced in the past 12 months, mean (SD)**		4.5 (5.4)
	Do not know, n (%)	23 (46)
	0-1, n (%)	9 (18)
	2-3, n (%)	6 (12)
	≥4, n (%)	9 (18)
**Symptoms experienced during CAD episodes (top 5)^b,c^, n (%)**		
	Fatigue or tiredness	39 (83)
	Increased weakness	32 (68)
	Decreased stamina	31 (66)
	Shortness of breath	31 (66)
	Dark urine	22 (47)
**Number of symptoms experienced during CAD episodes^c^, mean (SD)**		6 (2.9)
	1-2, n (%)	8 (17)
	3-4, n (%)	8 (17)
	5-6, n (%)	8 (17)
	>6, n (%)	23 (49)
**Triggering factors^b,c^, n (%)**		
	Cold temperatures	39 (83)
	Winter	32 (68)
	Air conditioning	26 (55)
	Sudden change in temperature	20 (43)
	Infection	15 (32)
	Psychological stress	10 (21)
	Surgery	6 (13)
	High humidity	3 (6)
	Other^d^	5 (11)
	None	3 (6)
**Number of factors that triggered CAD symptoms^c^, mean (SD)**		3.3 (1.8)
	<3, n (%)	16 (34)
	3-4, n (%)	19 (40)
	>4, n (%)	12 (26)

^a^CAD: cold agglutinin disease.

^b^Multiple-choice question.

^c^Sample size: n=47.

^d^Handling cold items (1/47, 2%), stress and physical activity (1/47, 2%), constant hemolysis creating chest pain in my sternum (1/47, 2%), overexertion (1/47, 2%), and unspecified (1/47, 2%).

### Impact of CAD on Patients’ Daily Life

When patients were asked in an open-ended question (“Which aspects of your daily life (emotionally, physically, socially, etc) are the most impacted by CAD?”), they named physical well-being (40 quotations), emotional well-being (31 quotations), and social life (28 quotations). Patients who reported an impact on their physical well-being primarily indicated the need to take naps (7 quotations), the inability to perform physical activities (7 quotations), and the need to limit their daily tasks (6 quotations). Emotional well-being was primarily affected owing to depression (7 quotations), frustration (6 quotations), or anxiety (4 quotations).

Among the 20% (10/50) of the patients who were employed at the time of the study, 90% (9/10) declared that CAD affected their professional life (Table 4). A total of 60% (30/50) of the patients reported that their household finances were affected by the disease. Totally, 83% (10/12) of the patients aged <60 years said that CAD affected their household finances, compared with 42% (8/19) of those aged >70 years. In total, 74% (37/50) of the patients had to cover out-of-pocket cost associated with the disease. Alternative medicines (21/50, 42%), transportation costs to medical appointments (16/50, 32%), and office visits or hospital care (16/50, 32%) were the most cited out-of-pocket costs to be covered, with an average of 2 out-of-pocket costs per patient (Table 4).

When patients were asked, with suggested items, which CAD-related symptoms had the greatest impact on their daily life, 90% (45/50) of the patients reported fatigue, 58% (29/50) of the patients reported shortness of breath, and 44% (22/50) of the patients reported joint pain, headaches, or acrocyanosis.

**Figure 2:**
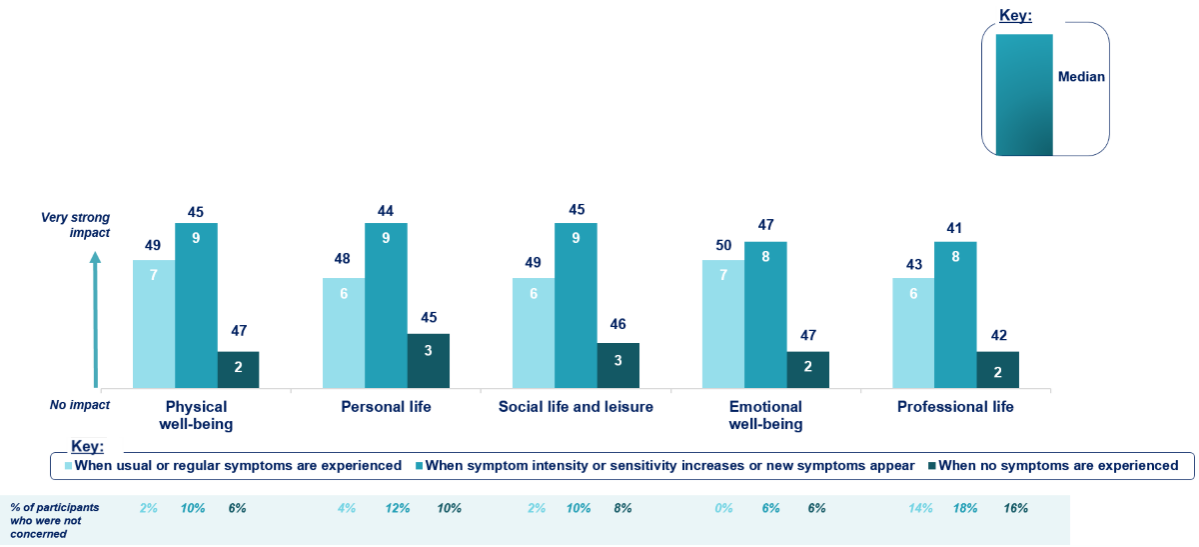
Impact of cold agglutinin disease on daily life reported by patients with that disease in the United States who were recruited for the web-based survey (N=50).

Patients were most affected during episodes with increased symptom intensity, increased sensitivity, or new CAD symptoms (median ≥8 out of 10, with 10=very strong impact), followed by episodes with usual symptoms (median ≥6 out of 10, with 10=very strong impact). Patients were less affected when no symptoms were experienced (median ≤3 out of 10), but they still indicated a negative impact on their quality of life (Figure 2).

**Table 4:** Impact of CAD^a^ on patients with CAD in the United States recruited for the web-based survey (N=50).

Impact of CAD		Values, n (%)
**Professional life^b,c^**		
	Had to take time off work	6 (60)
	Could not work as much as they would like to	5 (50)
	Not as efficient at work	2 (20)
	Concerned that they would not be able to do the job when applying for a new position	2 (20)
	Not able to work outside from late fall to early spring	1 (10)
	Had to make modifications to avoid handling cold items	1 (10)
	Unspecified	1 (10)
	Not affected	1 (10)
**Household finances**		
	No impact	20 (40)
	Mild impact	15 (30)
	Moderate impact	11 (22)
	Severe impact	3 (6)
	Not sure	1 (2)
**Out-of-pocket costs related to CAD**		
	Alternative medicines (eg, vitamins and herbal medicines)	21 (42)
	Transportation for medical appointments	16 (32)
	Office visits or hospital care	16 (32)
	Treatment for CAD and any related side effects	14 (28)
	Transportation to relocate to a warmer region	10 (20)
	Household services (eg, house cleaning and yard work)	9 (18)
	Supportive care (eg, psychological support and nutrition)	7 (14)
	Other^d^	5 (10)
	None	13 (26)

^a^CAD: cold agglutinin disease.

^b^Multiple-choice question.

^c^Sample size: n=10.

^d^Rental in warm area for 4 months each year (1/50, 2%); purchase more expensive health insurance (1/50, 2%); install a generator, which can be used if power is lost (1/50, 2%); move to a place for people aged >55 years, where everything is already available (1/50, 2%); and unspecified (1/50, 2%).

### Impact of CAD-Related Fatigue

**Figure 3:**
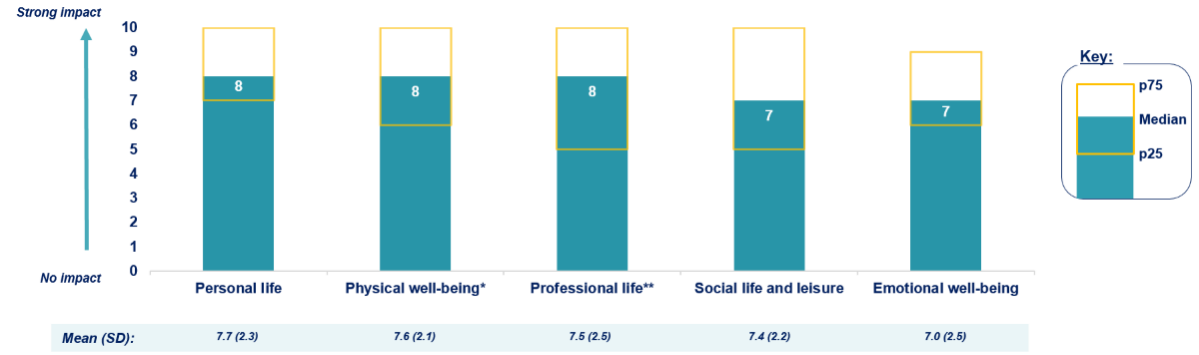
Impact of fatigue on daily life, reported by patients with cold agglutinin disease in the United States who were recruited for the web-based survey (N=50). p25: 25th percentile; p75: 75th percentile. *n=44 respondents affected; **n=37 respondents affected.

When patients who experienced fatigue, tiredness, lack of stamina, or weakness were asked in an open-ended question to describe how fatigue affected their daily life, they indicated that fatigue affected daily activities (38 quotations), associated fatigue with symptoms (37 quotations), and reported the need to implement solutions to cope with fatigue (31 quotations). Patients who reported fatigue-related symptoms primarily indicated weakness (22 quotations), difficulty in concentrating (6 quotations), and headaches (3 quotations). Those who indicated that fatigue affected their daily activities primarily cited an impact in terms of difficulty in completing daily tasks or starting new projects (12 quotations), difficulty in doing household chores (8 quotations), and the need to avoid physical activities (7 quotations). When asked on a scale from 0 to 10 (0=fatigue has no impact; 10=fatigue has a strong impact), it was found that fatigue had a significant impact on all aspects of daily life that were cited: personal life, physical well-being, professional life, social life and leisure, and emotional well-being (median ≥7 out of 10; Figure 3). Patients who experienced fatigue on a daily basis reported a more severe impact on each aspect of their daily life.

### Symptom Management Methods

In total, 92% (46/50) of the patients reported using a management method at the time of the survey. The management method most often reported was cold avoidance (41/50, 82%), followed by rituximab treatment (10/50, 20%) and blood transfusion (4/50, 8%). On average, patients reported using 1.4 (SD 0.79) management methods at the time of the survey.

The main management methods used by patients with CAD in the past were cold avoidance (41/50, 82%), rituximab treatment (23/50, 46%), and corticosteroid treatment (15/50, 30%). Before the survey, patients reported using an average of 2.1 (SD 1.2) management methods.

A total of 32% (16/50) of the patients had to relocate away from where they used to live because of their CAD. Of these 16 patients, 9 (56%) patients relocated for part of the year and 7 (44%) patients relocated permanently. Totally, 59% (20/34) of the patients who had not relocated owing to CAD said that they want to do so. Incidentally, patients who relocated owing to CAD were more satisfied with their current management methods than those who did not relocate (7/15, 47% and 9/31, 29%, respectively).

Only 24% (12/50) of the patients declared having received blood transfusions owing to CAD in the previous 12 months. Among these 12 patients, 6 (50%) patients received at least three blood transfusions in the previous 12 months. When asked about their level of satisfaction regarding blood transfusion, half of the patients were satisfied, whereas the other half were unsatisfied (median 5 out of 10, with 10=agree with the item “I am satisfied with the way blood transfusions are performed”). Patients also reported that they often required assistance with transportation for blood transfusion appointments (median 6.5 out of 10, with 10=agree with the item “Someone often has to take me to or from my blood transfusions”) or that the time spent in having infusions had an impact on their activities (median 5.5 out of 10 with 10=agree with the item, “Time spent for a blood transfusion has an impact on other activities”).

Overall, among the 92% (46/50) of the patients who reported using a management method at the time of the survey, only 34% (16/46) were satisfied with the method used, primarily because of the improvement in CAD symptoms (4 quotations)*.* A total of 31% (14/46) of the patients were dissatisfied with their current management method, including 11% (5/46) who were very dissatisfied. The most common reason for dissatisfaction was the lack of efficacy of the management method applied (5 quotations).

As CAD-related fatigue has a significant impact on the patients’ quality of life, approximately all patients who experienced fatigue (44/45, 98%) had devised a coping mechanism to manage the symptoms. However, only 11% (5/45) of the patients sought professional help for fatigue. On average, patients implemented 5 coping mechanisms to deal with this symptom. However, only 28% (12/44) of the patients were satisfied with the coping mechanism that was implemented, among whom only 5% (2/44) were very satisfied. In contrast, 32% (14/44) of the patients were dissatisfied, including 9% (4/44) patients who were very dissatisfied.

## Discussion

### Principal Findings

This study brings new evidence of the burden of CAD from patients’ perspectives.

In particular, our survey provided new data on fatigue experience (importance and type of fatigue). A total of 90% (45/50) of the patients reported having experienced fatigue (fatigue, tiredness, lack of stamina, or weakness). Fatigue was reported daily by 44% (20/45) of the patients, several times a week by 27% (12/45), and only after physical exertion by 16% (7/45) of the patients. When fatigue was experienced, patients mainly mentioned that it usually fluctuated during the course of the day. For example, in the survey, a patient stated the following:

Anything I need to routinely do, such as laundry, cooking, and light to moderate housework, leaves me completely drained.

Fatigue, which is common to many disorders, could also explain the long delay between the first symptoms and the initial diagnosis.

Our findings also reveal that CAD had a great impact on patients’ daily activities, physical well-being, emotional well-being, social life, and household finances. For example, a patient stated the following in the results:

I have always been a very active person. Not being able to do the same activities is very hard emotionally. We look normal to most people, but we are not and that explanation is very hard for most people to understand and I get frustrated trying to explain CAD to people.

In total, 38% (19/50) of the patients experienced at least one CAD episode in the 12 months before the study. On average, they experienced 4 to 5 episodes during this time. Patients reported that CAD episodes were most often triggered by cold temperatures, winter, and air conditioning.

Finally, new data on disease severity and progression have been obtained. Most patients (27/50, 54%) considered their disease to be moderate or severe. Fatigue was reported to be the most prominent and most impactful symptom. People who were employed indicated that their work was affected by CAD. In total, 42% (21/50) of the study group reported that their symptoms had worsened since the time of diagnosis. To reduce the impact of CAD symptoms, most patients (46/50, 92%) reported using a management method.

These results confirm and supplement the findings from other studies.

Su et al [12] interviewed patients with CAD using a semistructured interview guide and demonstrated that fatigue, tiredness, or lack of energy; reaction to cold environments; shortness of breath; and trouble thinking or concentrating were the main symptoms experienced by patients and described the impact of CAD on daily activities. Nonetheless, that study included a very limited number of patients (n=16). This study, involving a large group of patients and using a questionnaire built with a patient advocacy group, allowed us to confirm the main findings published by Su et al [12] and provided additional evidence of the burden of CAD.

In comparison with other studies, our results also confirm the great impact of CAD on daily activities [12]. Moreover, the triggers quoted by patients (cold temperatures, winter, and air conditioning) were consistent with the information found in other studies [17]. Finally, the management method most often reported was cold avoidance, which is consistent with previous studies [6,17,18]. However, CAD symptoms have been shown to vary for the first time in a patient survey, implying variation in the impact of CAD. In addition, lack of satisfaction with the management methods used, which was not assessed in the previous studies, was shown.

### Limitations

A few limitations of this study should be mentioned.

The self-administered web-based nature of the survey is likely to be biased toward patients who have access to the internet and are comfortable with using computers (ie, bias toward young patients). Approximately 10% of Americans do not use the internet [19], and many others may have access, but decline to participate in web-based surveys. This bias may partly explain the difference between the study group profile, who were younger in terms of average age at the time of diagnosis (59.2 years) and average age at the time of study (66.7 years), compared with those in the previous studies. A multinational observational study of 232 patients with CAD by Berentsen et al [8] indicated a mean age of 67 years at disease onset, mean age of 68 years at the time of diagnosis, and mean age of 72 years at the time of study. In addition, the men-to-women sex ratio in our study group (0.22) varied from that (0.56) in the study by Berentsen et al [8]. Therefore, the results are likely to be biased toward women’s perspective. It has been shown that this reflects the main characteristics of web-based users willing to share their experience with a disease [20].

Another potential limitation of a web-based survey is that it may exclude patients who are non–English-speaking or socioeconomically disadvantaged without internet access, who may have a different subset of symptoms, management methods, and diagnostic characteristics. In addition, if the information was not documented and the patient had to rely solely on memory to complete the questionnaire, certain clinical information may have been underestimated (eg, age at diagnosis, symptoms before diagnosis, number of CAD episodes over the past 12 months, symptoms during the episodes, and perception of disease severity). In addition, the study was only performed in the United States, and treatment, mental health, and so on can be different in other countries.

Patients were recruited from the CAD Unraveled website and CADF, which introduces a selection bias that may not be representative of the entire US population with the disease. This selection bias can be identified in the fact that the frequency of circulatory symptoms is lower in this study than in large studies among people with CAD [8,11]. However, our study population was different, as it was based in the United States (different states and temperature), with secondary CAD accepted. Collection of data was also different, as they were obtained from patients and not from physicians. The acrocyanosis reported in our study is only a part of the cold-induced circulatory symptoms. Other cold-induced circulatory symptoms are observed in CAD. For instance, in our study, 50% (25/50) of the patients reported Raynaud syndrome or acrocyanosis. Another selection bias can be the length of the survey. A total of 30 minutes were needed to complete the whole questionnaire, which could have affected the answer rate. Finally, among the 1155 patients reached, 50 (4.33%) patients completed the questionnaire and no hard criteria for diagnosis of CAD were used, which can bias the sample. The sample may not be representative of the entire US population with CAD. This participation rate can be explained by different factors (no compensation, methodology used, presence of caregivers or people not diagnosed with CAD or with invalid emails in the CAD Unraveled database, overlap between both databases, reduction of the open survey window, etc).

The absence of a question on race and ethnicity is a limitation of our study, as it has historically affected access to care in the United States. In addition, recall bias could have affected the results, especially for the CAD burden, as it was asked at 3 different time points. As many patients (36/50, 72%) were retired or did not work owing to reasons other than CAD, our assessment of the potential impact of CAD on professional life was limited. Circulatory symptoms were not fully addressed in the context of impact on daily life. However, such impact can be substantial.

Finally, even with the purpose of the study being scientifically driven with no patient compensation, voluntary participation, and review by an ethics committee, a bias in information cannot be ruled out, as most of the questions were closed (39/45, 87%), limited in spontaneous reporting, and completed knowing the study was conducted by a pharmaceutical company. In addition, the study may be biased as it was conducted by a pharmaceutical company and Carenity, a company closely linked to the pharmaceutical company conducting the study. However, a patient advocacy group dedicated to CAD was involved to reduce this bias as much as possible.

### Conclusions

In conclusion, our study provides new insights into the symptoms associated with CAD and the impact of the disease from the patient’s perspective. It particularly highlights the importance and type of fatigue, seriousness of the disease as perceived by patients, impact of CAD even when no symptoms are experienced, and importance of CAD symptom variation, implying that the impact of CAD varies over time. In addition, difficulties in CAD diagnosis have been raised. Patients with CAD experienced difficulty in receiving proper diagnosis and treatment owing to their rare disease; hence, they should be diagnosed earlier and, then, closely monitored and advised by a health care provider with knowledge of how to treat the disease.

Additional studies are necessary to better understand the burden of CAD-related symptoms, especially fatigue, and the patient’s needs regarding symptom management. It is also important to raise awareness among health care professionals regarding CAD-related symptoms to appropriately diagnose and support these patients. Specifically, health care professionals should proactively discuss ways to manage fatigue, which is not often discussed during consultations.

## Appendix

Multimedia Appendix 1 Symptoms experienced by patients before diagnosis (N=50).

## Abbreviations

AIHA: autoimmune hemolytic anemia
CAD: cold agglutinin disease
CADF: Cold Agglutinin Disease Foundation
