# Supplementary figures and images for: The Burden of Cold Agglutinin Disease on Patients’ Daily Life: Web-Based Cross-sectional Survey of 50 American Patients
Source: JMIR Form Res. 2022 Jul 22;6(7):e34248. doi: 10.2196/34248 (PMC9356335; doi:10.2196/34248)

**Supplemental Data**


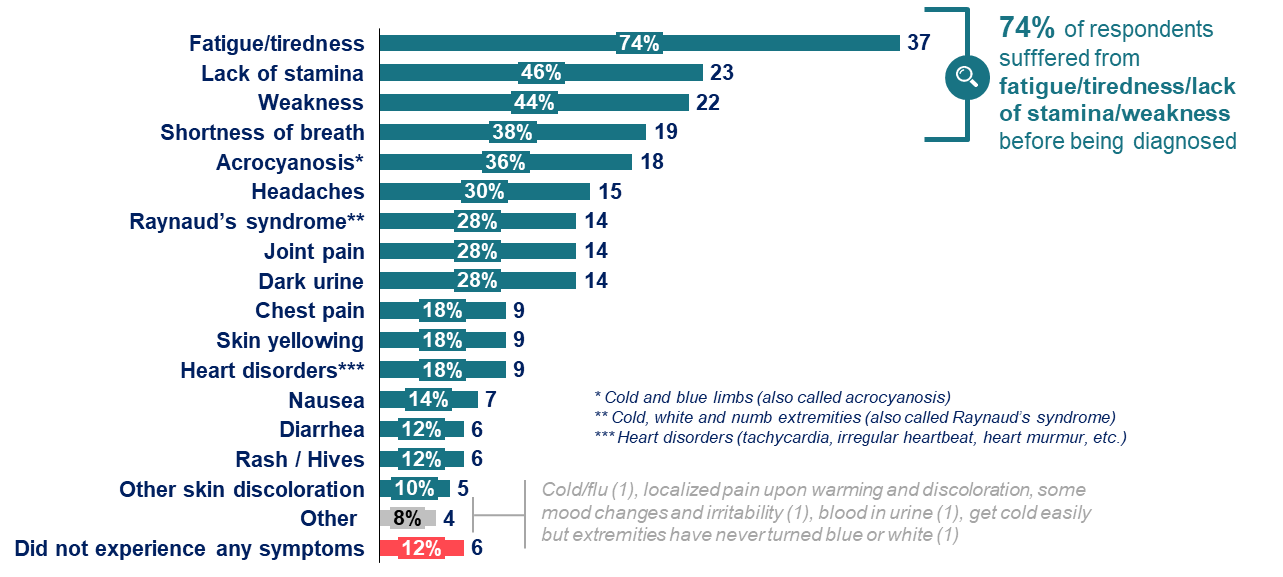


**Supplemental Figure 1. Symptoms experienced by patients before diagnosis (n = 50)**

Supplement: Multimedia Appendix 1 [file formative_v6i7e34248_app1.docx]
